# Myeloid Cell Modulation by Tumor-Derived Extracellular Vesicles

**DOI:** 10.3390/ijms21176319

**Published:** 2020-08-31

**Authors:** Ihor Arkhypov, Samantha Lasser, Vera Petrova, Rebekka Weber, Christopher Groth, Jochen Utikal, Peter Altevogt, Viktor Umansky

**Affiliations:** 1 Skin Cancer Unit, German Cancer Research Center (DKFZ), 69120 Heidelberg, Germany; ihor.arkhypov@gmail.com (I.A.); Samantha.Lasser@medma.uni-heidelberg.de (S.L.); Vera.Petrova@medma.uni-heidelberg.de (V.P.); Rebekka.Weber@medma.uni-heidelberg.de (R.W.); Christopher.Groth@medma.uni-heidelberg.de (C.G.); j.utikal@dkfz.de (J.U.); p.altevogt@dkfz.de (P.A.); 2 Department of Dermatology, Venereology and Allergology, University Medical Center Mannheim, Ruprecht-Karl University of Heidelberg, 68167 Mannheim, Germany

**Keywords:** extracellular vesicles, cancer, myeloid cells, immunosuppression

## Abstract

Extracellular vesicles (EV) can carry proteins, RNA and DNA, thus serving as communication tools between cells. Tumor cells secrete EV, which can be taken up by surrounding cells in the tumor microenvironment as well as by cells in distant organs. Tumor-derived EV (TEV) contain factors induced by tumor-associated hypoxia such as heat shock proteins or a variety of microRNA (miRNA). The interaction of TEV with tumor and host cells can promote cancer angiogenesis, invasion and metastasis. Myeloid cells are widely presented in tissues, comprise the majority of immune cells and play an essential role in immune reactions and tissue remodeling. However, in cancer, the differentiation of myeloid cells and their functions are impaired, resulting in tumor promotion. Such alterations are due to chronic inflammatory conditions associated with cancer and are mediated by the tumor secretome, including TEV. A high capacity of myeloid cells to clear EV from circulation put them in the central position in EV-mediated formation of pre-metastatic niches. The exposure of myeloid cells to TEV could trigger numerous signaling pathways. Progenitors of myeloid cells alter their differentiation upon the contact with TEV, resulting in the generation of myeloid-derived suppressor cells (MDSC), inhibiting anti-tumor function of T and natural killer (NK) cells and promoting thereby tumor progression. Furthermore, TEV can augment MDSC immunosuppressive capacity. Different subsets of mature myeloid cells such as monocytes, macrophages, dendritic cells (DC) and granulocytes take up TEV and acquire a protumorigenic phenotype. However, the delivery of tumor antigens to DC by TEV was shown to enhance their immunostimulatory capacity. The present review will discuss a diverse and complex EV-mediated crosstalk between tumor and myeloid cells in the context of the tumor type, TEV-associated cargo molecules and type of recipient cells.

## 1. Introduction

### 1.1. Extracellular Vesicles

Initially thought to eliminate unneeded cell compounds, extracellular vesicles (EV) are now recognized as means of intracellular communication [1]. Current understanding of EV biogenesis enables their classification into exosomes and microvesicles, providing specific markers for EV characterization [1,2]. Exosomes are considered as smaller (50–150 nm) EV that originate from the endosomal system; thus, tetraspanins such as cluster of differentiation (CD)9, CD63, CD81 are used as exosome markers [1,3]. Microvesicles are larger (50–1000 nm) EV that shed directly from the cellular membrane, and, therefore, annexin A1 was suggested as a marker for microvesicles [1,2,3]. In addition to the differences in generation, EV subtypes show differential distribution of cargo molecules such as proteins, RNA and DNA that has been comprehensively reviewed elsewhere [1,2]. According to the guidelines proposed by the International Society for Extracellular Vesicles, the current review will use the definition “extracellular vesicles” since the final consensus on specific EV markers has not yet been reached [4].

In view of the function of EV as mediators of intercellular communication, an explicable interest is rising regarding the EV-mediated triggering of phenotypical changes in target cells, which can be induced by surface signaling and/or the uptake of EV [5]. Membrane-associated proteins, lipids or sugars on EV can interact with surface molecules of target cells, triggering intracellular signaling cascades and mediate the internalization of EV, in which lectin family receptors, adhesion molecules and numerous other receptor-ligand interactions are involved [5,6,7]. Moreover, EV are also considered to carry so called “surface cargo” of adsorbed plasma-derived molecules: immunoglobulins, complement proteins, cytokines, coagulation factors, enzymes, thiols, lipoproteins, DNA modulating the surface EV-cell interactions [8,9]. Although it is technically challenging to prove, which molecule is incorporated into EV membrane and which is absorbed, the diversity of EV surface components highlights the importance of surface interactions between EV and recipient cells. 

The involvement of membrane lipid rafts in the function of EV is less well studied. The rafts can activate signaling pathways or perform sorting functions by regulating proteins associated with them [10]. It has been reported that EV express increased levels of certain lipids and thus differ from the cellular membrane of cells, from which they are derived [11]. Moreover, the regulation of the PI3K/Akt pathway by lipid rafts through the participation of insulin receptors or endothelial growth factor receptors, which are transported by EV, was already reported [10]. However, the biological functions of lipids found in EV have hardly been investigated.

Various studies have shown that microRNA (miRNA) essentially contribute to EV function and that their function can be lost when miRNA is depleted by knocking down crucial enzymes of miRNA biogenesis [12,13,14]. Since miRNA and other non-coding RNA play a pivotal role in EV-mediated cancer-host cell interaction [15], we provide in this review an insight into miRNA biogenesis and sorting into EV. EV contain large numbers of RNA that is intact and functional in recipient cells [16]. During the generation of EV, cytosolic RNA (miRNA, other small non-coding RNA, long non-coding RNA, mRNA, tRNA, and rRNA) are taken up into the lumen [16,17]; this could be confirmed by the observation that intact RNA can still be isolated from EV treated with RNAse. The RNA profile of EV often does not reflect that of the original cells, suggesting that the RNA recruitment into EV is a selective process [16]. Since EV secreted by different cell types share an overlapping set of miRNA, the existence of a common mechanism for selective miRNA export is conceivable [18]. However, the exact process by which miRNA is sorted into EV is unknown. Several studies suggest an involvement of RNA-binding proteins in the packaging of miRNA into EV by recognizing specific short miRNA motifs [19,20,21,22,23,24]. Post-transcriptional modifications of RNA seem to play a crucial role in this process [23,25].

### 1.2. Myeloid Cells

**Figure 1:**
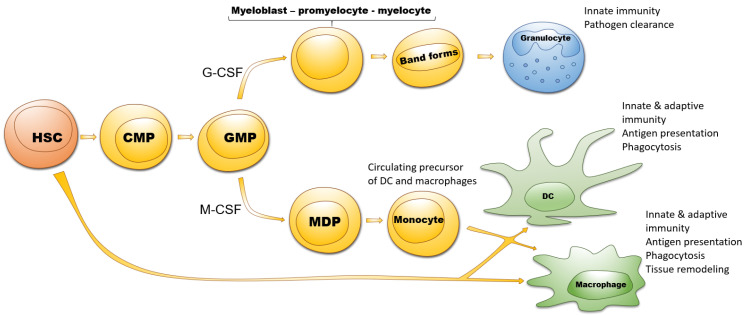
Differentiation of myeloid cells. Hematopoietic stem cells (HSC) differentiate into the common myeloid progenitors (CMP) and further into the granulocyte-macrophage progenitors (GMP). The granulocyte colony-stimulating factor (G-CSF) generates the differentiation of GMP via myeloblasts, promyeloblasts, myelocytes, and band forms into granulocytes (shown by the arrows), whereas macrophage colony-stimulating factor (M-CSF) induces GMP development towards monocytes, macrophages or DC via monocyte/macrophage and dendritic cell progenitors (MDP) (shown by the arrows).

Myeloid cells differentiate from hematopoietic stem cells (HSC), represent the majority among immune cells and include monocytes, macrophages, DC and granulocytes [26]. HSC differentiate into common myeloid progenitors followed by granulocyte-macrophage progenitors (GMP) (Figure 1) [27]. Granulocytes (neutrophils, basophils and eosinophils) are developed from GMP under the influence of granulocyte colony-stimulating factor (G-CSF) through the stages of myeloblasts, promyelocytes, myelocytes, metamyelocytes and band cells. Monocytes and DC develop from GMP via monocyte/macrophage and DC progenitors induced by the macrophage colony-stimulating factor (M-CSF) [27].

Terminally differentiated macrophages, granulocytes and DC play a major role in the immune defense against pathogens and in the tissue remodeling [26,27]. However, myeloid cells can be reprogrammed to promote tumor progression and angiogenesis [28,29]. Under the conditions of chronic inflammation or cancer, GM-CSF, G-CSF, M-CSF, tumor necrosis factor (TNF)-α, interleukin (IL)-1β, IL-6, transforming growth factor (TGF)-β and IL-10 are constantly secreted, leading to the alteration of myelopoiesis and generation of myeloid-derived suppressor cells (MDSC) [30,31,32]. CCL2-dependent recruitment of inflammatory monocytes (Ly6C^hi^CD11b^+^CD11c^–^MHCII^–^VCAM1^–^CCR2^+^) to the cancer site and their exposure to M-CSF and other tumor-derived factors leads to the generation of tumor-associated macrophages (TAM) that demonstrate self-renewal capacity [29,33] Both MDSC and TAM are key players in cancer progression and will be discussed in this review. 

In addition to these soluble factors, abundant EV production by tumor cells contributes to the modulation of host immune cells during cancer progression [34]. Macrophages are known to perform clearance of circulating EV [35,36,37]. Furthermore, fully differentiated cells like macrophages and mature DC were demonstrated to uptake EV more efficiently than monocytes or immature DC [38]. Taking into account the importance of EV for tumor development and metastasis and a high capacity of myeloid cells for EV internalization, the current review will discuss various aspects of myeloid cell modulation by TEV, leading to immune escape. 

## 2. Tumor-Derived Extracellular Vesicles (TEV)

Tumors secrete EV, which can be locally taken up by neighboring cancer cells and cells from the tumor microenvironment promoting cancer growth [34]. Tumors can also release EV into the circulation and promote metastasis formation at distant organs [34]. These systemic TEV effects were shown to be organ-specific and dependent on the integrin expression on TEV [39]. 

In comparison to EV secreted by normal cells, TEV present a similar morphology but a distinct molecular composition, which reflects the stressed state of tumor cells [40]. Additionally, tumor cells secrete EV to a higher extent than normal cells [40]. Being one of the hallmarks of cancer, hypoxia largely accounts for these properties of TEV. Hypoxia and acidic pH lead to cellular stress in tumors and can induce increased release of EV with altered miRNA, protein and lipid cargo, promoting cancer angiogenesis, invasion and metastasis [41,42,43,44]. Moreover, TEV composition and secretion can be modulated by anti-cancer treatment [45].

Since EV can carry biomarkers that are relevant for tumor diagnostics, they are widely studied as a liquid biopsy source [46]. The identification of TEV among the whole EV population isolated from the peripheral blood or other liquids from cancer patients requires the recognition of specific tumor markers in the respective EV fractions. To meet these needs, several approaches to EV isolation and characterization are suggested. Recent advances have been made for the isolation of TEV in melanoma, when antibodies against melanoma antigen chondroitin sulfate proteoglycan (CSPG)4 were used for on-beads immunocapturing of melanoma-derived EV [47]. Size fractionation might aid identification of tumor-specific EV by reducing their heterogeneity [48]. For instance, human epidermal growth factor receptor 2, a marker of breast cancer, has been demonstrated to be stronger expressed in EV with the size of 20–100 nm, while prostate-specific membrane antigen (PSMA), a prostate cancer marker, was higher in the EV fraction of 200–1000 nm [48].

An analytical microfluidic platform has been developed for the isolation of tumor-specific EV-RNA, achieving 94% specificity and 10-fold yield increase as compared to other methods [49]. Simultaneous analysis of exosome markers (CD9, CD63 and CD81) with PSMA in nanoscale flow cytometry has been shown to be a useful method for characterizing the origin of TEV in prostate cancer [50]. Surface-enhanced Raman spectroscopy (SERS) nanotags enabled EV phenotyping by using simultaneously anti-CD63-conjugated magnetic beads and specific detection antibody-coated SERS nanotags [51]. Since heat-shock protein (HSP)70 is highly expressed in cancer cells and is associated with a worse prognosis, prospective clinical trial was initiated to study the capability of HSP70 as TEV biomarker [52]. This study has demonstrated that the HSP70 level in TEV is higher in metastatic than non-metastatic patients and that is inversely correlated with the therapy response.

Delivery of TEV to the immune cells can lead to various dysfunctions, causing tumor immune escape [53]. TEV can directly inhibit effector immune cells [54] or elicit their protumorigenic effects by modulating myeloid cells [55]. In contrast, TEV as carriers of tumor antigens might trigger an anti-tumor immune response via the transfer of tumor antigens to DC [56]. Therefore, understanding the complexity of EV-mediated crosstalk between tumor and myeloid cells is crucial for restoring and maintaining an efficient anti-tumor immune response. 

## 3. EV in Crosstalk between Tumor Cells and Myeloid Progenitors

The model for the compartment of myeloid cells has been recently revised by Bassler et al. [26] and suggested to be rather a “motorway junction” on a hematopoietic “autobahn” than a “trunk of a tree”. The new model highlighted the ability of myeloid progenitors to dynamically respond to extracellular signals during stress, inflammation etc. Moreover, single-cell RNA-sequencing analysis indicates that HSC and progenitor cells exhibit an early precommitment for developing into distinct cell types [26,57]. Such precommitment becomes more pronounced during aging resulting in a shift from the lymphatic towards the myeloid compartment [58] that is in agreement with immunosuppression accompanying cancer development in elderly patients. 

Immature myeloid progenitor cells are continuously generated in the bone marrow under physiological conditions [32]. They show no suppressive potential against natural killer (NK) and T cells and normally differentiate to mature myeloid cells; however, in cancer, their normal differentiation is affected. In that case, immature myeloid progenitor cells may obtain immunosuppressive capacity typical for MDSC [59]. EV can contribute here in several ways. Thus, an uptake of acute myeloid leukemia-derived EV, expressing c-Myc, by myeloid progenitor cells resulted in the selective proliferation of MDSC [60]. Moreover, it has been demonstrated that EV derived from Lewis lung carcinoma or breast cancer cells block the differentiation of myeloid progenitors into DC in vitro [61,62]. Melanoma-derived EV have been shown to transfer MET kinase to circulating vasculogenic and hematopoietic myeloid progenitor cells in vivo, enhancing bone marrow mobilization [63]. This transfer led to an increased formation of lung and bone metastases. Patients with metastatic melanoma showed increased total MET and phospho-MET levels in circulating EV. Moreover, these patients had elevated MET expression in circulating CD45- C-KITlow/+ tyrosine kinase with immunoglobulin-like and EGF-like domains (TIE)2+ bone marrow progenitor cells [63]. Furthermore, a direct immunosuppressive capacity towards effector T cell was obtained by murine immature myeloid cells through melanoma EV-mediated programmed death ligand (PD-L)1 upregulation via Toll-like receptor (TLR) signaling [64]. The upregulation of PD-L1 by myeloid and tumor cells induces T cell anergy through the interaction with PD-1 on T cells and represents a critically important immune escape mechanism shared by different cancers [65]. The blocking of PD-1/PD-L1 axis by anti-PD-1 antibodies significantly increased the efficiency of cancer immunotherapy [65].

## 4. EV in Crosstalk between Tumor Cells and Monocytes

Monocytes represent a subset of myeloid cells circulating in the peripheral blood and migrating into tissues during inflammation [66]. They play a key role in phagocytosis, tissue homeostasis and resolving overactivated immune responses, preventing thereby a tissue damage [66]. Monocytes have a high plasticity to differentiate into macrophages or DC, but this plasticity is either altered or reduced in cancer [67]. Being constantly produced by tumor cells, released into the peripheral blood and taken up by monocytes, TEV contribute to these changes. In fact, treatment of CD14+ monocytes with melanoma-derived or colorectal carcinoma-derived EV impaired their ability to differentiate into DC [68]. Furthermore, upon the exposure to melanoma TEV, monocytes change their cytokine secretion profile, upregulate immunosuppressive markers and gain an ability to inhibit the function of effector immune cells [69]. 

TEV derived from different cancer types share some similarity and induce the production of IL-6 [64,68,70,71], IL-10 [64,72,73,74], TNF-α [64,68,71], TGF-β, IL-1β, chemokine (C-C motif) ligand (CCL)2 and CCL4 [64,68,70,71] by monocytes. It has been demonstrated that IL-6 production triggered by TEV via TLR2 and TLR4 is needed for the activation of signal transducer and activator of transcription (STAT)3 [71]. TGF-β produced by monocytes upon treatment with TEV derived from melanoma or colorectal carcinoma cells was shown to mediate an inhibition of T cell activity [68]. 

Several groups have reported an upregulation of PD-L1 and downregulation of human leukocyte antigen (HLA)-DR in monocytes treated with TEV. In one study, melanoma-derived EV have been demonstrated to skew monocyte differentiation towards suppressive CD14+HLA-DR-/low cells, which also lose the expression of costimulatory molecules CD80 and CD86 [68]. Huber et al. [69] found that a special set of miRNA (miRNA-146a, -155, -125b, -100, -125a, -146b, -99b and let-7e) is responsible for HLA-DR downregulation, secretion of IL-6 and CCL2 by monocytes upon the exposure to melanoma EV, leading to the inhibition of T cell effector functions. Furthermore, melanoma-derived EV were found to convert monocytes into immunosuppressive MDSC via PD-L1 upregulation triggered by the signaling via the HSP86/TLR4/Nuclear factor (NF)-κB axis [64]. In chronic lymphocytic leukemia (CLL), the transfer of noncoding Y RNA hY4 to monocytes resulted in the upregulation of PD-L1 in a TLR7-dependent manner [70]. In addition, this study demonstrated that targeting TLR7 in vivo led to the reduction of tumor load. Moreover, glioblastoma stem cell-derived EV have been shown to upregulate PD-L1 on monocytes and skew them towards M2-like cells associated with the phosphorylation of STAT3, p70S6 kinase and extracellular signal-regulated kinase (Erk)1/2 [75]. In contrast, Lorgulescu et al. [76] stated that glioma-derived EV were not capable of inducing PD-L1 expression in peripheral monocytes. However, they caused a significant downregulation of HLA-DR expression. Pancreatic cancer-derived EV could downregulate HLA-DR, induce arginase-1 (Arg-1) expression, increase production of reactive oxygen species (ROS) and the levels of phosphorylated STAT1 and STAT3 in monocytes [77]. 

It has been demonstrated an acquisition of a tumor-associated macrophages (TAM)-like phenotype by monocytes exposed to TEV. The interaction between EV secreted by head and neck cancer cells (PCI-1 cells) and monocytes has been shown to activate the NF-κB pathway resulting in a TAM-like phenotype [78]. Hypopharyngeal carcinoma cells (FaDu cells) overexpressing the transcription factor Snail, a key inducer of epithelial to mesenchymal transition (EMT), have been shown to secrete miRNA-21-enriched EV, which increased the expression of M2 markers (CD163, CD206) and the pro-angiogenic ability of CD14+ monocytes [73]. Wang et al. [79] observed that monocytes exposed to gastric cancer-derived EV develop into PD-1+ macrophages, producing M2-like cytokines and inducing the suppression of CD8 T cell activity. Interestingly, a study with EV from poorly metastatic melanoma cells noted that these EV could stimulate an innate immune response and block metastasis into the lungs [80]. When mice were preconditioned with such EV, they developed significantly less melanoma metastases. The study showed further that this effect was mediated by the pigment epithelium-derived factor expressed in EV delivered to non-classical Ly6C^low^ monocytes. Therefore, existing evidence underlines the acquisition of immunosuppressive potential by monocytes upon the exposure to TEV, which was dependent on the metastatic potential of the EV-producing cancer cells. 

## 5. EV in Crosstalk between Tumor Cells and Macrophages

Macrophages are terminally differentiated tissue-resident cells derived from yolk-sac embryonic precursors and replenished by circulating monocytes during inflammation [26]. Macrophages show high plasticity and constantly adapt to environmental changes by modifying their functional state. The main functions of macrophages include elimination of infectious agents, promotion of wound healing and regulation of adaptive immunity [81]. Two types of macrophages are described. M1 macrophages express high levels of IL-1β, IL-6, IL-12, IL-23, and TNF-α (pro-inflammatory cytokines), ROS, nitric oxide (NO), and low levels of IL-10 and TGF-β (anti-inflammatory cytokines). They are activated by interferon (IFN)-γ or bacterial lipopolysaccharides (LPS) and play a role in the elimination of tumor cells. In contrast, M2 macrophages express high levels of IL-10 and low levels of IL-12, are activated by IL-4, IL-10, IL-13 and glucocorticoid hormones and promote tumor progression [82,83]. TAM have characteristics similar to M2 macrophages. They produce CCL22, prostaglandin E2 and TGF β, leading to immunosuppression [59]. Similar to MDSC, TAM express PD-L1 and Arg-1, causing T cell anergy and apoptosis [84,85]. Using these mechanisms, TAM promote tumor growth, metastasis and angiogenesis, suppress anti-tumor immunity and protect tumor cells from chemotherapy-induced apoptosis [86,87]. Furthermore, their accumulation has been shown to be associated with a poor clinical outcome [88]. Since macrophages clear EV from systemic circulation as mentioned above, these cells could be an important target of TEV.

EV are known to facilitate pre-metastatic niche formation by polarizing macrophages towards M2 phenotype in ovarian cancer [89]. Initiation of pre-metastatic niche formation by TEV has also been shown in pancreatic cancer by the stimulation of TGF-β production by Kupffer cells and of fibronectin production by hepatic stellate cells, leading to the recruitment of bone marrow-derived macrophages [90].

Growing evidences suggested a role of TEV in regulating the polarization of macrophages [91]. Thus, EV produced by colorectal cancer and melanoma cells were reported to induce mixed M1 and M2 polarization and cytokine production [74,92,93]. Breast cancer-derived EV have been demonstrated to induce an IL-6-mediated pathway via gp130/STAT3 signaling, leading to TAM polarization [94]. Hypoxia-induced melanoma TEV have been demonstrated to promote M2 polarization of macrophages, to transfer let-7a miRNA and increase tumor growth [95]. Cheng et al. [96] stated that TEV derived from liver cancer cells can upregulate PD-L1 expression as well as cytokine secretion by macrophages through STAT3 signaling, and that the treatment with melatonin modulated the function of TEV, leading to the attenuation of immunosuppressive capacity of macrophages.

Extensive data show the involvement of miRNA in the TEV-mediated modulation of macrophage phenotype. In hypoxic glioma-derived EV, miRNA-1246 was found to be the most enriched miRNA. The transduction of macrophages with miRNA 1246 resulted in the activation of STAT3 and the inhibition of NF-κB signaling, leading to the skewing of macrophages towards a tumorigenic phenotype in vitro and in vivo [97]. Similarly, human macrophages transduced with miRNA-222-3p, which is significantly increased in EV isolated from serum of patients with epithelial ovarian cancer, upregulated the expression of genes linked to M2 polarization and showed increased IL-10 secretion in vitro [98]. In addition, miRNA-222-3p caused the downregulation of suppressor of cytokine signaling (SOCS)3 in these macrophages, induced STAT3 signaling and a TAM-like phenotypic polarization. As a result, miRNA-222-3p transfected macrophages promoted the growth of ovarian cancer cells in vitro and in vivo [98].

A study with hepatocellular carcinoma (HCC)-derived EV indicated that miRNA-146a 5p from EV contributed to M2-like macrophage polarization, and that the suppression of Sal-like protein (SALL)4, a transcription factor for miRNA-146-5p, reversed T cell exhaustion induced by these macrophages in vivo [99]. Furthermore, miRNA-150 found to be present in TEV accumulated in the plasma of HCC patients, promoted tumor development through increasing vascular endothelial growth factor (VEGF) secretion by TAM [100]. The inhibition of miRNA-150 in tumor-bearing mice suppressed VEGF secretion and tumor growth in this study. Additionally, the delivery of miRNA-21 to tumor-associated macrophages led to increased xenograft tumor progression in mice [73].

In another study, the delivery of miRNA-23a by EV derived from liver cancer cells to macrophages led to the upregulation of PD-L1 expression via a Phosphatidylinositol 3-kinase (PI3K)/Protein kinase Akt pathway resulting in the attenuation of CD8 T cell functions [101]. In contrast, the treatment of murine 4T1 breast cancer cells with epigallocatechin gallate induced the secretion of miRNA-16 containing EV, which inhibited TAM infiltration and M2-like polarization by the suppression of NF-κB activation [102]. Taken together, macrophages are highly affected by TEV, leading to protumor effects in most cases, although the exact outcome of this interaction should be related to the content of TEV and the type of cancer cells producing these EV.

## 6. EV in Crosstalk between Tumor Cells and Dendritic Cells

DC are terminally differentiated myeloid cells that present antigens to T cells and play a pivotal role in crosslinking innate and adaptive immunity [103]. During normal hematopoiesis, DC originate from HSC, whereas under inflammatory conditions, they arise from monocytes [26,103]. DC need a contact with bacteria, viruses or damaged tissues to be activated and can promote the differentiation of naïve T cells, inducing an immune response [104]. Mature DC infiltrate tumors and recruit anti-tumor effector immune cells [105]. However, in cancer, VEGF, M-CSF, IL-6, adenosine and hypoxia accumulated in the tumor microenvironment affect DC functions, leading to the enrichment of immature, functionally incompetent DC [106]. This results in a weak stimulation of the anti-tumor immune response and accumulation of immunosuppressive MDSC [107]. In contrast to monocytes, which are more likely exposed to TEV in the circulation, DC contact these vesicles in the tumor microenvironment and draining lymph nodes [108,109,110].

It has been observed that TEV derived from murine thymoma and melanoma cell lines are internalized by DC in the skin and trafficked to the draining lymph node, resulting in the inhibition of DC maturation and TGF-β production [111]. Besides, melanoma TEV have been shown to induce IL-6 production in DC, leading to STAT3-dependent matrix metalloproteinase 9 production by tumor cells, promoting thereby their invasiveness [112]. Such effect on DC was demonstrated to be TLR2/4-dependent and mediated by HSP72 and HSP105 expressed on the TEV surface. In vitro DC treatment with EV from lung cancer cells caused downregulation of CD80, CD86 and major histocompatibility complex (MHC)-II, whereas immunosuppressive markers including PD-L1 were upregulated [61]. In this study, the authors also found that Lewis lung carcinoma-derived EV inhibited DC migration to draining lymph nodes, downregulating chemokine receptors and reducing antigen-specific CD4 T cell proliferation in a PD-L1-dependent manner. The delivery of miRNA-212-3p by pancreatic cancer-derived EV led to the inhibition of the expression of RFXAP, a transcription factor for MHC-II, resulting in the downregulation of MHCII in vitro [113]. The authors concluded that TEV, containing miRNA-212-3p, induced tolerogenic DC. In another study, miRNA-203 delivered by pancreatic cancer-derived EV mediated the downregulation of TLR4 expression on DC [114].

In contrast to the above described inhibition of DC immunostimulatory activity, TEV were also reported to convey tumor antigens to DC resulting in the activation CD8 T cell anti-tumor response [56]. Two studies demonstrated that TEV loaded with miRNA-155, miRNA 142 or let 7i by electroporation could promote the maturation of DC, causing an increase in T cell proliferation and cytotoxicity against tumor target cells in vitro [115,116]. Moreover, HCC-TEV improved DC therapeutic efficiency resulting in the elevation of CD8 T cell numbers, an increase in IFN-γ production and a decrease in the production of IL-10 and TGF β [117]. Furthermore, TEV-pulsed DC were shown to reduce numbers of regulatory T cells (Treg) and augment the therapeutic effect of antibodies against PD-1 and sorafenib in an HCC mouse model [118]. Therapeutic administration of TEV-loaded DC also showed a beneficial effect alone and in combination with MDSC depletion in pancreatic cancer [119].

## 7. EV in Crosstalk between Tumor Cells and Granulocytes

Granulocytes are myeloid cells, containing granules with defensive factors and enzymes, which play an important role in the innate immune response [120]. Granulocytes can be divided into three subsets: neutrophils, basophils and eosinophils. Neutrophils play a critical role in cancer [121]. On one hand, they show a strong cytotoxic activity and have the ability to recruit other immune cells due to chemokine release [122]. On the other hand, similar to monocytes and macrophages, neutrophils show a great plasticity and develop protumorigenic features under the exposure to TGF-β, G-CSF and IFN-β, promoting tumor angiogenesis and metastasis [121,123]. Furthermore, a high neutrophil-to-lymphocyte ratio was found to be associated with decreased overall survival (OS) of patients in different types of cancer [124]. The infiltration of the tumor with mature neutrophils was proposed to be a prognostic factor for tumor recurrence [125,126].

Eosinophils are also known to play a role in cancer development. An accumulation of eosinophils has been observed in the tumor tissue and peripheral blood of cancer patients [127]. Moreover, this was reported to be associated with a beneficial OS and progression-free survival (PFS) in melanoma patients [128,129]. 

During infection, neutrophils form neutrophil extracellular traps (NET) to eliminate pathogens. However, in cancer, NET formation could contribute to cancer-associated thrombosis, leading to thromboembolic complications [130,131]. Interestingly, breast cancer-derived EV were demonstrated to induce NET formation in neutrophils and enhance thrombosis when administrated in vivo [132]. 

TEV could also influence the phenotype of neutrophils. Thus, colorectal cancer stem cell (CRCSC)-derived EV increased the survival of neutrophils and endowed neutrophils with a protumor phenotype that was mediated by IL-1β [133]. In contrast, different populations of myeloid progenitors were not affected by CRCSC-derived EV in vivo, suggesting that this effect on neutrophils was not dependent on the proliferation of myeloid progenitors [133]. Furthermore, Cheng et al. [134] reported a correlation between the abundance of the major miRNA in CRCSC-derived EV, miRNA-146a, and increased numbers of tumor-infiltrating neutrophils. 

Neutrophils could be polarized towards a protumorigenic phenotype upon the exposure to gastric cancer-derived EV [135]. High mobility group box (HMGB)1 was found to be a key factor transported by gastric cancer-derived EV that triggers TLR4 signaling followed by the activation of NF-κB and protumor activation of neutrophils [135].

Data on TEV impact on eosinophils and basophils are relatively scarce. It was found that EV derived from epithelial lung cancer cells contain γ-glutamyl transpeptidase 1 and contributed to the conversion of leukotriene C4 produced by eosinophils into leukotriene D4, which is known to mediate a number of protumor effects [136].

## 8. EV in Crosstalk between Tumor Cells and MDSC

MDSC represent a heterogeneous population of myeloid cells generated under chronic inflammatory conditions or cancer and mediate a profound immunosuppression in the tumor microenvironment [32]. In mice, two MDSC subsets have been described: CD11b+Ly6G-Ly6Chigh monocytic (M-MDSC) and CD11b+Ly6G+Ly6Clow polymorphonuclear (PMN-MDSC). Human MDSC could be divided into three subpopulations: CD11b+CD14+HLA-DRlow/-CD15- M-MDSC, CD11b+CD14-HLA-DRlow/-CD15+ PMN-MDSC and CD11b+CD14-HLA-DRlow/-CD15- early (e)MDSC [137]. MDSC are known to strongly inhibit the anti-tumor activity of T and NK cells through different mechanisms. They express PD-L1 and Fas antigen ligand, inducing T cell anergy and apoptosis, increase the production of immunosuppressive adenosine, ROS, NO and expression of Arg-1, leading to the down-regulation of T cell receptor ζ-chain expression as well as stimulate Treg generation [138,139,140]. In melanoma patients, the high frequency of MDSC has been shown to correlate with an advanced stage, disease progression, decreased OS and PFS and with low response to immunotherapy [141,142]. 

Several studies found that HSP triggered TLR signaling, leading to the increase of MDSC immunosuppressive potential. Diao et al. [143] have demonstrated that renal cell carcinoma cells produce TEV containing HSP70 that stimulate the TLR2/Myeloid differentiation primary response protein MyD88 (MyD88)/STAT3 signaling in MDSC, leading to tumor progression. HSP70 has been shown to be expressed on TEV isolated from patients with breast, pulmonary or ovary cancer to a higher extent than in EV derived from healthy donors [144]. In line with these data, renal cancer TEV showed abundant expression of HSP70. Therefore, blocking EV-HSP70 with A8 peptide prevented its interaction with TLR2 and blocked EV-mediated activation of MDSC. Moreover, A8 treatment potentiated 5-FU therapy since the latter enhanced the HSP70 expression on EV [144]. Triggering of the TLR2/MyD88 signaling pathway by TEV derived from thymoma, mammary carcinoma or colon carcinoma cells and containing HSP72, has been shown to induce IL-6 production by MDSC and autocrine activation of STAT3 [145]. Another study reported that TEV induced IL-6 release from MDSC and postulated that the role of TLR2 signaling could be dependent on the type of TEV-producing cancer cells [146]. Comparing the effects of EV derived from melanoma cells in vitro and in vivo, they found that only EV isolated from in vitro cultured tumor cells exerted a TLR2-dependent effect on MDSC in their system. Furthermore, it has been demonstrated that the inactivation of prostaglandin E2 and TGF-β blocks MDSC induction via TEV derived from mammary tumor cells in vivo [147]. The authors suggested that in vivo MDSC generation was dependent on the delivery of prostaglandin E2 and TGF-β by TEV. It was earlier shown that the expression of receptors for prostaglandin E2 and the activation of MDSC was supported by such interactions [148]. 

TEV have been shown to transfer functional RNA from cancer cells to MDSC [149]. In oral squamous cell carcinoma (OSCC), hypoxic TEV enhanced the suppressive effect of MDSC on γδ T cells through a miRNA-21/Phosphatase and tensin homolog (PTEN)/PD-L1 axis [150]. This study further showed that targeting miRNA-21 in combination with anti-PD-1 therapy had an anti-tumor effect on OSCC-bearing mice. Hypoxia-induced glioma-derived EV have been demonstrated to induce MDSC via miRNA-10a and miRNA-21 since MDSC from TEV-treated mice showed an enhanced suppressive effect on CD8 T cells that was attenuated by inhibiting miRNA-10a or miRNA-21 [151]. A similar effect has been shown to be mediated by the transfer of miRNA-29a and miRNA-92a in hypoxia-induced glioma TEV [152]. Interestingly, these miRNA promoted proliferation of MDSC, while miRNA-92a also enhanced their immunosuppressive function. Bruns et al. [153] stated that a transfer of miRNA-155 by CLL-derived EV contributed to MDSC induction, which was prevented by vitamin D, repressing the expression of miRNA-155 in CLL cells. Gastric cancer-derived EV were demonstrated to deliver miRNA-107 to MDSC, leading to their expansion and an upregulation of Arg-1 expression [154].

Not only cancer cells themselves but also tumor-educated mesenchymal stem cells (MSC) could modulate MDSC promoting breast cancer progression [155]. MSC-derived EV, containing TGF-β, complement component C1q and semaphorins could enhance immunosuppressive capacity of M-MDSC via PD-L1 upregulation and drive their differentiation towards M2-macrophages, showing an increased Arg-1 activity and IL-10 secretion [155]. Therefore, EV derived from cancer and stroma cells could support tumor development through the activation of MDSC.

## 9. Conclusions

**Figure 2:**
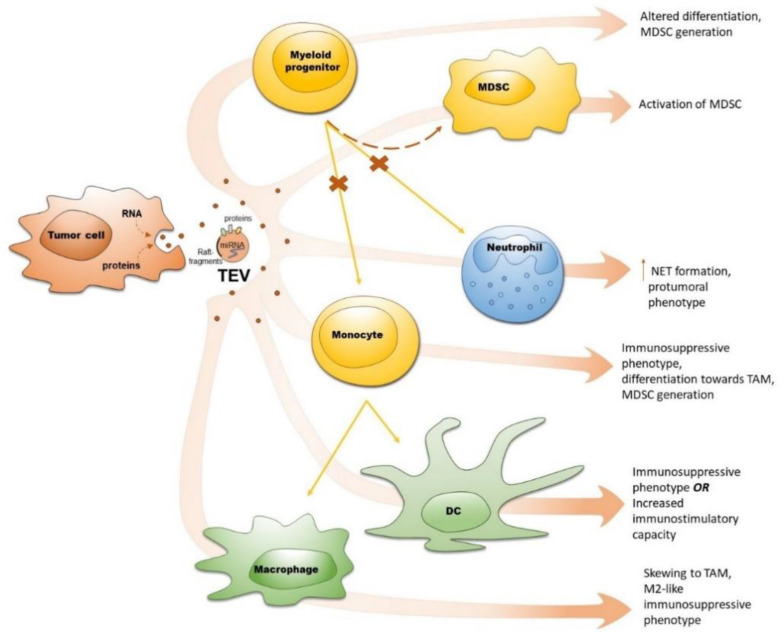
Tumor-derived extracellular vesicles (TEV) affect myeloid cell differentiation and function. Tumor cells secrete TEV containing tumor-derived factors (proteins, mRNA, miRNA etc.), which interact with myeloid progenitors (partially blocking their differentiation to mature myeloid cells that is shown with “x”), monocytes, neutrophils and myeloid-derived suppressor cells (MDSC), resulting in tumor-promoting immunosuppression (shown with arrows). Exposure of dendritic cells (DC) to TEV leads to either acquisition of immunosuppressive function or induction of immunostimulatory capacity (shown with arrows).

TEV are transferred to myeloid cells and modulate their differentiation and phenotype to induce the formation of pre-metastatic niches at distant sites further supporting tumor progression (Figure 2). Such TEV capacity is attributed to their cargo molecules (oncogenes, various HSP, numerous miRNA etc.), which could trigger surface signaling or exert intracellular effects after being internalized (Table 1). Sorting of these cargo molecules into TEV is regulated by tumor-associated hypoxia [41,42,43,44,95,97,150,151,152] and cytotoxic chemotherapy [45]. Exposure of monocytes to TEV induced their conversion into immunosuppressive MDSC [102], whereas DC could acquire immunostimulatory capacity under the influence of TEV containing tumor-associated antigens [56,115,116,117,118,119], leading to anti-tumor effects. Therefore, the state of cells producing TEV, cargo of TEV and state of recipient cells are the factors, determining the outcome of TEV-mediated crosstalk between tumors and myeloid cells. 

Numerous studies implicate EV as a delivery system for anti-cancer drugs or vaccine vectors to improve an anti-cancer immunity [156,157]. Other therapeutic strategies aim at disrupting EV-mediated protumorigenic effects by interfering with the secretion, uptake and signaling of EV [40]. Among them are anti-CD9 and anti-CD63 antibodies, which induced macrophage-dependent depletion of circulating EV [158]. This approach showed a therapeutic benefit in mice with breast tumors based on the prevention of metastasis without affecting primary tumor growth [158]. In addition, inhibiting TEV-associated miRNA with anti-miRNA oligonucleotides was demonstrated to reduce the growth of human oral squamous cell carcinoma cells in nude mice [159]. Therefore, a combination of blocking immunoinhibitory effects of TEV and EV application as an immunostimulatory vector could become an effective anti-cancer therapy.

## Figures and Tables

**Table 1:** Effects of TEV on myeloid cells.

Cell Types	TEV-Associated Molecules	Effects	Sources of TEV	References
myeloid progenitors	c-Myc	accumulation of MDSC	human cell line of myeloid leukemia; patient-derived cells	[60]
	not shown	block of differentiation, accumulation of MDSC	murine cell lines of lung cancer and breast cancer	[61,62]
	MET kinase	activation of the Erk pathway, pro-metastatic behavior	murine cell line of skin cancer	[63]
	HSP90α	TLR4-dependent MDSC induction	murine and human cell lines of skin cancer	[64]
monocytes	not shown	immunosuppressive phenotype	human cell lines of colorectal cancer, skin cancer, pancreatic cancer, gastric cancer and malignant brain tumor	[68,75,76,77,79]
	Y RNA hY4	activation of TLR7-dependent signaling, protumorigenic phenotype	human cell line of lymphocytic leukemia	[70]
	unknown proteins	activation of TLR-dependent signaling	amniotic fluid and malignant ascites from patients with ovarian cancer	[71]
	hyaluronan	activation of PI3K/Akt signaling, production of anti-inflammatory cytokines	human cell line of pancreatic cancer	[72]
	miRNA-21	immunosuppressive phenotype	human cell line of head and neck cancer	[73]
	not shown	contact time-dependent effect (early contact: immunosuppressive; late contact: proinflammatory)	human cell line of colon cancer	[74]
	set of miRNA	conversion of monocytes into MDSC	murine and human cell lines of skin cancer	[69]
	not shown	secretion of pro-inflammatory cytokines	human cell line of head and neck cancer	[78]
	pigment epithelium-derived factor	cancer cell clearance at the pre-metastatic niche	murine and human cell lines of skin cancer	[80]
macrophages	macrophage migration inhibitory factor	TGF-β-dependent formation of pre-metastatic niches	murine and human cell lines of pancreatic cancer	[90]
	not shown	altered secretion of cytokines	murine cell lines of skin cancer; human cell lines of colorectal cancer and liver cancer	[92,93,96]
	gp130	cytokine secretion, induction of STAT3 signaling	murine cell line of breast cancer	[94]
	let-7a, chemoattractants	macrophage recruitment, immunosuppressive phenotype	murine cell line of skin cancer under hypoxic conditions	[95]
	miRNA-1246	immunosuppressive phenotype, induction of STAT3 signaling, increased motility of glioma cells	human cell line of glioma under hypoxic conditions	[97]
	miRNA-222	immunosuppressive phenotype, induction of STAT3 signaling	human cell line of ovarian cancer	[98]
	miRNA-146a	immunosuppressive phenotype, induction of STAT3 signaling	murine and human cell lines of liver cancer	[99]
	miRNA-150	upregulation of VEGF, immunosuppressive phenotype	HEK cells, overexpressing miR-150	[100]
	miRNA-23a	immunosuppressive phenotype, activation of PI3K/Akt signaling	human cell line of liver cancer	[101]
DC	not shown	inhibition of DC maturation, production of anti-inflammatory cytokines	murine cell lines of thymic cancer and skin cancer	[111]
	HSP72, HSP105	TLR signaling-dependent matrix metallopeptidase 9 production	murine cell lines of skin cancer and breast cancer; tumor tissue from patients with breast cancer	[112]
	not shown	immunosuppressive phenotype, decreased migration of DC to the draining lymph nodes	murine cell lines of lung cancer and breast cancer	[61]
	miRNA-212-3p	downregulation of MHCII expression	human cell line of pancreatic cancer	[113]
	miRNA-203	downregulation of TLR4 expression	human cell line of pancreatic cancer	[114]
	miRNA-155, miRNA-142 or let-7i	enhanced maturation of DC and increased stimulation of T cells	murine cell lines of breast cancer and colon cancer	[115,116]
	tumor antigens	decreased Treg, increased survival of mice treated with sorafenib and anti-PD-1 antibodies	murine cell line of liver cancer	[118]
	not shown	increased survival of mice	murine cell line of pancreatic cancer	[119]
Granulocytes	not shown	induction of NET formation by neutrophils	murine cell line of breast cancer; plasma from breast tumor-bearing mice	[132]
	not shown	increased neutrophil survival, protumorigenic phenotype	murine cell line of colorectal cancer	[133]
	miRNA-146a	increased neutrophil infiltration of tumors	human cell line of colorectal cancer; serum from patients with colorectal cancer	[134]
	HMGB1	TLR4-dependent NF-κB activation, increased autophagic response	human cell line of gastric cancer	[135]
	γ-glutamyl transpeptidase 1	conversion of eosinophil-derived leukotriene C4 into leukotriene D4	human cell line of lung cancer	[136]
MDSC	HSP70	TLR2 signaling-dependent cytokine release and induction of STAT3, activation of MDSC, tumor progression	murine cell lines of kidney cancer, skin cancer and colon cancer; human cell lines of colon cancer, prostate cancer, cervix cancer	[143,144]
	HSP72	TLR2 signaling-dependent induction of STAT3, activation of MDSC	murine cell lines of colon cancer, lymphatic cancer; tissue from tumor-bearing mice (breast cancer, skin cancer, lymphoma); human cell line of lung cancer	[145,146]
	miRNA-21	PTEN-dependent PD-L1 upregulation, enhanced suppression of γδ T cell functions	human cell line of oral cancer under hypoxic conditions	[150]
	miRNA-10a, miRNA-21, miRNA-29a, miRNA-92a	expansion and activation of MDSC	murine cell line of glioma under hypoxic conditions	[151,152]
	miRNA-155	activation of MDSC	human cell line of lymphocytic leukemia	[153]
	miRNA-107	expansion of MDSC with increased Arg-1 expression	human cell line of gastric cancer, serum from patients with gastric cancer	[154]
	TGF-β, C1q, semaphorins	upregulated PD-L1 expression, differentiation towards M2 macrophages, tumor progression	human and murine MSC isolated from breast tumors	[155]
	prostaglandin E2 and TGF-β			

## Abbreviations

Arg-1	Arginase-1
CCL	Chemokine (C-C motif) ligand
CD	Cluster of differentiation
CLL	Chronic lymphocytic leukemia
CSPG	Chondroitin sulfate proteoglycan
CRCSC	Colorectal cancer stem cell
DC	Dendritic cells
EMT	Epithelial to mesenchymal transition
Erk	Extracellular signal-regulated kinase
EV	Extracellular vesicles
G-CSF	Granulocyte colony-stimulating factor
GMP	Granulocyte-macrophage progenitors
HCC	Hepatocellular carcinoma
HLA	Human leukocyte antigen
HMGB1	High mobility group box 1
HSC	Hematopoietic stem cells
HSP	Heat-shock protein
IFN	Interferon
IL	Interleukin
LPS	Lipopolysaccharides
M-CSF	Macrophage colony-stimulating factor
MDSC	Myeloid-derived suppressor cells
MHC	Major histocompatibility complex
miRNA	MicroRNA
M-MDSC	Monocytic MDSC
MSC	Mesenchymal stem cells
MyD88	Myeloid differentiation primary response protein MyD88
NET	Neutrophil extracellular traps
NF	Nuclear factor
NK	Natural Killer
NO	Nitric oxide
OS	Overall survival
OSCC	Oral squamous cell carcinoma
PD	Programmed cell death protein
PD-L	Programmed death ligand
PFS	Progression-free survival
PI3K	Phosphatidylinositol 3-kinase
PMN-MDSC	Polymorphonuclear MDSC
PSMA	Prostate-specific membrane antigen
PTEN	Phosphatase and tensin homolog
ROS	Reactive oxygen species
SALL	Sal-like protein
SERS	Surface-enhanced Raman spectroscopy
SOCS	Suppressor of cytokine signaling
STAT	Signal transducer and activator of transcription
TAM	Tumor-associated macrophages
TIE	Tyrosine kinase with immunoglobulin-like and EGF-like domains
TEV	Tumor-derived extracellular vesicles
TGF	Transforming growth factor
TLR	Toll-like receptor
TNF	Tumor necrosis factor
Treg	Regulatory T cells
VEGF	Vascular endothelial growth factor
